# Losartan-Induced Angioedema: A Case Report

**DOI:** 10.7759/cureus.37818

**Published:** 2023-04-19

**Authors:** Henry Mann, Sagar Pandey, Sindhu Pokhriyal, Josef Kusayev, Alix Dufresne

**Affiliations:** 1 Internal Medicine, One Brooklyn Health System, Interfaith Medical Center, New York City, USA; 2 Medicine, New York Medical College, Valhalla, USA; 3 Cardiology, One Brooklyn Health System, Interfaith Medical Center, New York City, USA

**Keywords:** arb, angioedema, losartan, drug-induced angioedema, losartan induced angioedema

## Abstract

Angiotensin-converting enzyme (ACE) inhibitors and angiotensin receptor blockers are both classes of drugs used in the management of hypertension, heart failure, chronic kidney disease, and proteinuria. While angioedema induced by ACE inhibitors has been well-documented, angioedema induced by angiotensin receptor blockers (ARBs) has not. We present the case of losartan-induced angioedema requiring tracheostomy in a 48-year-old African American male. To our knowledge, there have only been twenty case reports published to date about losartan-induced angioedema. Although in the immediate short-term, our patient made a complete recovery, he had a sudden cardiac arrest a few months after the incident of angioedema and died.

## Introduction

Angioedema is defined as the localized swelling of subcutaneous and submucosal tissue caused by the extravasation of fluids into the interstitial space [1]. Angiotensin receptor blockers (ARBs) selectively block the binding of angiotensin II to the angiotensin type 1 receptor. ARBs inhibit the renin-angiotensin-aldosterone system and are used for hypertension, heart failure, chronic kidney disease, and proteinuria. Their effect is similar to angiotensin-converting enzyme (ACE) inhibitors; however, they are associated with fewer side effects (like angioedema) since they do not directly interfere with bradykinin breakdown by angiotensin-converting enzyme [2]. Multiple studies have reported half the rate of angioedema with angiotensin receptor blockers as compared to ACE inhibitors, specifically 0.1% [1]. ARBs likely cause angioedema by upregulation of angiotensin type 2 receptors secondary to the blockage of angiotensin type 1 receptors [1]. This paper will discuss a case of angioedema induced by losartan, an ARB; there have been only twenty case reports published to our knowledge documenting losartan-induced angioedema.

## Case presentation

**Figure 1:**
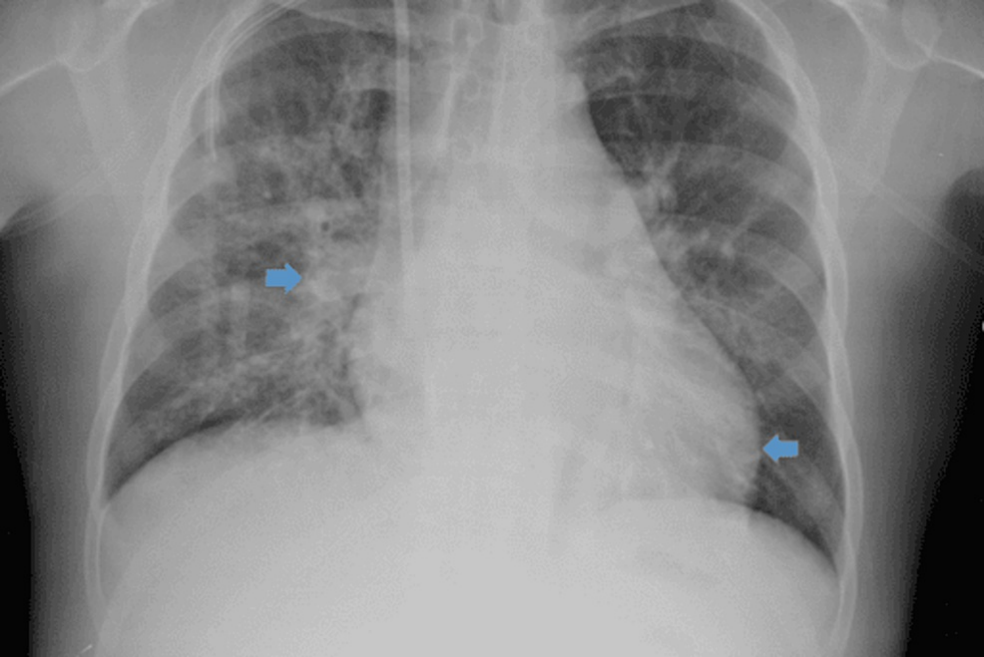
Chest X-ray on admission Chest X-ray reveals mild cardiomegaly and right perihilar edema.

Our patient is a 48-year-old African American male with a past medical history of end-stage renal disease on dialysis, acute pancreatitis, diabetes mellitus, and hypertension, who was admitted to the hospital with dizziness and leg swelling for two days after missing a dialysis session. His history was also significant for smoking a pack a day of cigarettes for twenty years and drinking a pint of hard liquor a day for an unknown duration. He was reported to be allergic to seafood and iodine. Physical exam on admission showed only 1+ pitting edema and a right chest Permacath in place. Remarkable labs on admission can be found in the table below (Table 1). Chest X-ray (Figure 1) on admission showed mild cardiomegaly and perihilar edema bilaterally.

**Table 1:** Patient's laboratory findings on admission

Laboratory test	Results	Normal range
White blood cells	4.9 cells/µL	4,500 - 11,000 cells/µL
Hemoglobin	10.0 g/dL	11.0 - 15.0 g/dL
Hematocrit	30.3%	35 - 46%
Mean corpuscular volume	76.1 fL	80 - 100 fL
Platelets	192 PLT/µL	130,000 - 400,000 PLT/µL
Aspartate aminotransferase	20 U/L	5 - 34 U/L
Alanine transaminase	19 U/L	10 - 55 U/L
Total bilirubin	0.3 mg/dL	0.2 - 1.2 mg/dL
Blood urea nitrogen	25.7 mg/dL	9.8 - 20.1 mg/dL
Creatinine	3.08 mg/dL	0.57 - 1.3 mg/dL
Estimated glomerular filtration rate	24.2 mL/min/1.73m^2^	>=90.0 mL/min/1.73m^2^
Potassium	4.4 mmol/L	3.5 - 5.1 mmol/L
Phosphorus	4.9 mg/dL	2.3 - 4.7 mg/dL
Magnesium	1.8 mg/dL	1.6 - 2.6 mg/dL
Brain natriuretic peptide	861 pg/mL	10.0 - 100.0 pg/mL
High sensitivity troponin	8.4 ng/L	0.0 - 17.0 ng/L
Prothrombin time	16.2 sec	9.8 - 13.4 sec
International normalized ratio	1.42 ratio	0.85 - 1.15 ratio
Partial thromboplastin time	36.0 sec	24.9 - 35.9 sec
Thyroid-stimulating hormone	1.98 µIU/ml	0.465 - 4.680 µIU/ml
Free thyroxine	1.66 ng/dL	0.78 - 2.19 ng/dL

On admission, the patient was on hydralazine 50 milligrams orally every eight hours, labetalol 200 milligrams orally every eight hours, and clonidine 0.2 mg orally as needed for his hypertension. Six hours after admission, his blood pressure was found to be 175/95 mmHg, and he was started on nifedipine 60 milligrams orally daily. On day two of admission, the patient underwent dialysis with the removal of 2.5 liters over three hours through his right Permacath; he tolerated the dialysis well. The patient was commenced on losartan 50 milligrams orally daily on day three of admission, as his mean arterial pressure was still persistently higher than 135 mmHg. An hour after the patient was given the first dose of losartan, he started complaining of voice changes, neck swelling, and sore throat. Losartan was discontinued. Three hours after this, his blood pressure dropped to 78/53 mmHg and then to 52/45 mmHg. A central line was immediately placed, vasopressors were started, and the patient was shifted to the intensive care unit. Epinephrine, methylprednisolone, diphenhydramine, and racepinephrine were also given, but the patient continued to deteriorate. In view of worsening face/neck swelling and difficult intubation, the patient underwent tracheostomy intubation. Over the next three days, he continued to improve on ventilatory and pressor support. CH50, C3, and C4 levels were normal, while C1 esterase inhibitor levels were high. By day six of admission, the patient was weaned off of vasopressors and was extubated. Post-extubation, the patient was commenced on a short course of steroids to reduce laryngeal swelling. The patient also failed a few swallowing tests but was eventually restarted on a complete renal diet a few weeks after extubation. Unfortunately, the patient experienced a sudden cardiac arrest a few months after this incident of angioedema and died.

## Discussion

Angioedema is defined as the localized swelling of subcutaneous and submucosal tissue caused by the extravasation of fluids into the interstitial space [1]. Broadly, the entity is classified as idiopathic angioedema without a discernible cause, histamine-mediated angioedema, or bradykinin-mediated angioedema [3]. 

ACE inhibitors are most commonly associated with the development of angioedema which occurs primarily due to the inhibition of the breakdown of bradykinin by ACE. The resulting accumulation of bradykinin triggers a downstream cascade via bradykinin 2 receptors and the release of substance P, which causes vasodilation and seepage of fluid into the interstitial space, ultimately leading to angioedema [1]. However, angioedema due to ARBs and its likely mechanism has been scarcely reported in the literature. A randomized crossover trial reported a two-fold increase in bradykinin levels following losartan administration. It was hypothesized that ARBs indirectly reduced ACE activity by increasing the stimulation of angiotensin type 2 receptors. This was because angiotensin type 1 receptor blockade by ARBs led to increased angiotensin II levels which in turn led to increased stimulation of angiotensin type 2 receptors, thus down-regulating ACE activity; down-regulation of ACE consequently results in the accumulation of bradykinin [4, 5]. 

One hour after our patient received the first dose of losartan, he started complaining of voice changes, neck swelling, and sore throat; after three hours, his blood pressure dropped. The temporal association of the administration of losartan and the development of angioedema helps to establish the role of losartan in the development of his angioedema. While anaphylaxis to losartan can also be argued to be behind the development of his cardiorespiratory symptoms, the delay in the onset of his symptoms and absence of urticaria/pruritus decreases the likelihood of losartan allergy as an explanation. On the contrary, the development of angioedema secondary to ARBs has been shown to have a time frame ranging from hours to days to months [6-10]. Hereditary angioedema was also ruled out in our patient following normal levels of CH50, C3, C4, and high levels of C1 esterase inhibitor. In addition, the possibility of angioedema induced by dipeptidyl peptidase inhibitors and neprilysin inhibitors was ruled out by the lack of these medications in the patient's medication history [11]. Lastly, the absence of an infectious prodrome of fever, lymphadenopathy, and sore throat in our patient effectively ruled out infectious causes of angioedema like deep neck space infections. 

## Conclusions

Though angioedema due to ACE inhibitors has been well documented, angioedema due to ARBs like losartan has not. In our case report, we document a case of angioedema due to losartan requiring tracheostomy while effectively ruling out other causes. To our knowledge, there have only been twenty case reports published to date about losartan-induced angioedema. ARB-induced angioedema, while not fatal in this case, is a serious adverse effect that can quickly lead to death. It is therefore important for clinicians to be wary of switching patients with angioedema from an ACE inhibitor to an ARB; it is also important to educate patients of the same effect.
